# Current Hospital Topics

**Published:** 1907-05-04

**Authors:** 


### CURRENT HOSPITAL TOPICS.

### National Hospital for the Paralysed and Epileptic.

A good deal of energy has been put into the administration of this hospital in recent years. The work of the institution is confined to a class of cases which unfortunately has shown a marked tendency to increase in recent years. The healthy are too apt to regard paralysis as a disease outside their immediate concern, but the increasing strain upon individual workers in large cities in the present day should make every thoughtful person take an interest in this hospital, and contribute something towards its support. The authorities have wisely invited its governors and supporters to supply the names and addresses of ladies and gentlemen who they may consider likely to become interested, if made acquainted with the pressing needs and extensive work of the National Hospital for the Paralysed and Epileptic. We are glad to note that His Majesty the King became an annual subscriber of ten guineas during the year 1906. There has been a saving in expenditure of £2 6s. 3d. per bed, a fact upon which we congratulate the administrative staff. The school of massage is a success, and the school of electrical treatment instituted during the year has made good progress, 35 certificates having been granted. The income of this hospital would appear to be over £3,000 less than the expenditure, a fact which we commend to those who have money to contribute for hospital purposes.

### Infectious Diseases in Private Families.

The London Fever Hospital has for over 100 years fulfilled a useful purpose by readily admitting patients suffering from infectious disease, with or without payment. A brougham ambulance with attendants, including a nurse where necessary, is sent to convey the patient to the hospital. It is interesting to know that during 1906 420 cases of scarlet fever were under treatment, with a mortality of 1.6 per cent.; 107 cases of measles were under treatment, with a mortality of 3.9 per cent.; and 46 cases of diphtheria were admitted, the mortality being 4.8 per cent. There were two cases of scarlet fever and two of measles amongst the nursing staff, whilst one wardmaid contracted diphtheria. The scarlet fever wards on the male side have been rebuilt, and these new wards have been occupied since June 1906. It is hoped to rebuild the same section of wards on the female side during the current year. The Committee record their satisfaction with the admirable manner in which Mr. Keith Young, the architect, has carried out the work of construction, owing to whom the work of reconstruction has been carried out with comparatively little interference with the work of the institution. It will take £20,000, of which about £12,000 has yet to be raised, to defray the cost of the rebuilding of the entire hospital. The London Fever Hospital provides accommodation for those who do not wish to go to the rate-supported hospitals of the Metropolitan Asylums Board, and there can be no doubt that Sir Shirley Murphy is right in stating that this hospital, by receiving cases of this type, has prevented a great deal of infection being spread abroad. Amongst the patients admitted to treatment were 49 nurses, 44 domestic servants, 25 pupils in schools, and ten patients from other hospitals. Lord Balfour of Burleigh contends that these figures prove that in a place like London the London Fever Hospital is a downright necessity for the safety of the people.

### The Royal Sea Bathing Hospital.

The report of this institution for the year 1906 contains a number of illustrations, which should enable the work to be much more fully appreciated by the subscribers than formerly. This hospital does good service for poorer Londoners, but, as the report of the visiting surgeons shows, the institution has not been utilised during 1906 to anything like the full extent of its possibilities. It is matter for regret that the number of gland and joint cases, admitted in an early stage of disease, still forms an exceedingly small proportion of the whole. The majority of the cases admitted had previously been under treatment, chiefly in metropolitan hospitals, and the visiting surgeons maintain that, had they been transferred to the Sea Bathing Hospital at the outset, the expenditure of both time and funds would have been lessened. It is desired to make it widely known that the Royal Sea Bathing Hospital is a place for the prevention of cripples by surgical treatment, under the best possible conditions, a fact the public and the profession should realise without delay. It is stated that, the results attained by Dr. Baily, in the treatment of disease by bacterial vaccines controlled by the opsonic index, confirm the view already held by the profession that, in carefully selected cases, this treatment may prove a valuble addition to our therapeutic resources. Five hundred and eighty-eight in-patients were under treatment during the year, the average daily number of patients being 133. The average residence per patient was 82.6 days. We are of opinion that this hospital should make the facilities it has to offer much more widely known. It should prove profitable, too, to put more energy into the management, with the object of obtaining increased publicity for the special work of the hospital, and increased support, in contributions, from the giving public.

